# Integrated surveillance and early warning system of emerging infectious diseases in China at community level: current status, gaps and perspectives

**DOI:** 10.1016/j.soh.2024.100102

**Published:** 2024-12-30

**Authors:** Chenjia Zhou, Suping Wang, Chenxi Wang, Ne Qiang, Leshan Xiu, Qinqin Hu, Wenyu Wu, Xiaoxi Zhang, Lefei Han, Xinyu Feng, Zelin Zhu, Leilei Shi, Peng Zhang, Kun Yin

**Affiliations:** a School of Global Health, Chinese Center for Tropical Diseases Research, Shanghai Jiao Tong University School of Medicine, Shanghai 200025, China; b School of Public Health, Shanghai Jiao Tong University School of Medicine, Shanghai 200025, China; c Discipline Planning Office, Shanghai Jiao Tong University School of Medicine, Shanghai 200025, China; d Institute of Medical Education, Shanghai Jiao Tong University School of Medicine, Shanghai 200025, China; e National Institute of Parasitic Diseases, Chinese Center for Disease Control and Prevention, Chinese Center for Tropical Diseases Research, WHO Collaborating Centre for Tropical Diseases, National Center for International Research on Tropical Diseases, Ministry of Science and Technology, National Health Commission Key Laboratory of Parasite and Vector Biology, Shanghai 200025, China; f Department of Engineering, School of Engineering, Computing, and Mathematics, College of Charleston, Charleston, SC 29424, United States; g Department of Pharmacology and Chemical Biology, Shanghai Jiao Tong University School of Medicine, Shanghai 200025, China; h Songjiang Research Institute, Shanghai Key Laboratory of Emotions and Affective Disorders (LEAD), Songjiang Hospital Affiliated to Shanghai Jiao Tong University School of Medicine, Shanghai 201699, China

**Keywords:** Emerging infectious diseases, Surveillance and early warning system, One Health, China, Community level

## Abstract

Emerging infectious diseases (EIDs) pose a significant threat to public health. Effective surveillance and early warning systems that monitor EIDs in a timely manner are crucial for their control. Given that more than half of EIDs are zoonotic, traditional integrated surveillance systems remain inadequate. Despite recent advances in integrated systems in China, there are few systemic reviews on the integrated surveillance and early warning system of EIDs at community level, particularly under the One Health framework. Here, this systematic review summarizes the current status of surveillance advances in China, including the multi-trigger integrated monitor system. It also highlights the mechanisms for embedding the One Health approach into local policy and practice, while identifying challenges and opportunities for improvement. Additionally, guidelines and recommendations are proposed to optimize the integration of multi-sectoral, multi-level and interdisciplinary cooperation at the human–animal–environment interface.

## 1 Introduction

Emerging infectious diseases (EIDs) are diseases that have recently appeared in humans or animals, often showing an increase in prevalence or expanding geographic distribution [1]. In recent decades, EIDs have frequently emerged worldwide, posing a significant threat to human health, animal populations, and ecosystems [2]. For instance, the 2003 outbreak of severe acute respiratory syndrome (SARS) in Guangdong Province, China resulted in 8422 cases and 919 deaths within just a few months [3]. The Zika virus, transmitted primarily by *Aedes* mosquitoes, has also raised international concern [4]. Most notably, the coronavirus disease 2019 (COVID-19) pandemic, caused by SARS-CoV-2 has resulted in millions of cases and deaths globally, demonstrating the profound societal, psychological and economic impacts of EIDs. Therefore, it is of vital importance to establish an integrated surveillance and early warning system with rapid response capabilities to address the challenges posed by EIDs.

Surveillance and early warning systems are the first and indispensable measures for effectively mitigating and eradicating the risks associated with serious infectious diseases. These systems are fundamental to managing emerging infectious threats, protecting public health and reducing the societal impact of disease outbreaks. By monitoring disease trends, detecting case clusters and conducting epidemiological investigations, surveillance systems enable the prompt detection of disease outbreaks and the improvement of response strategies. Currently, China has established a reporting system, a multi-level network involving various government agencies, medical institutions, laboratories and research organizations, covering 84,000 healthcare facilities nationwide [5]. Continuous surveillance is conducted throughout the year for major infectious diseases such as plague, poliomyelitis, malaria, and influenza [6].

The One Health approach offers critical opportunities to mitigate the impact of emerging diseases and reduce their future occurrence through increased cross-sectoral collaboration [7]. By adopting a One Health framework, China's surveillance systems can be more flexible and adaptive to infectious disease challenges. Surveillance data in China is sourced from multiple channels, including notifiable diseases, syndromes, all-cause mortality and laboratory surveillance. Effective integration of these data requires collaboration across multiple sectors at the community level, such as human health, animal health, and environmental health departments, as well as partnerships with hospitals, schools, and local communities [8]. Currently, the China CDC operates five general and four specific infectious disease surveillance systems. Despite significant progress, data quality, coherence of regional reporting and gaps in coverage of rural and remote areas remain a major challenge. In addition, transparency and timely dissemination of information are crucial for an effective response to public health emergencies and for international cooperation. In particular, surveillance systems for animals, especially wildlife, remain inadequate and pose a major challenge for effective early detection and warning of EIDs [9].

In this review, we systematically summarize the integrated surveillance and early warning system for EIDs in China at the community level, thereby addressing a gap in this field. The current system has numerous problems, such as the lack of information interoperability, inadequate communication channels, the inability to create an efficient overarching framework and the lack of an effective system for detecting multiple triggers and early warning. We highlight the current status of China's surveillance systems on human, environmental and wildlife health sectors. In addition, the gaps and challenges of the current integrated surveillance systems have been analyzed, which contribute to provide strategies aimed at fostering collaboration between different departments to effectively mitigate the risks posed by EIDs at the human–animal–environment interface. These guidelines aim to improve China's integrated surveillance system for EIDs, enabling early warning and more effective decision-making.

## 2 Current status

### 2.1 Technique development

The complicated and dynamic interactions among humans, animals and the environment are blurring traditional boundaries and significantly increasing the risk of EIDs. The incorporation of cutting-edge technologies such as remote sensing, metagenome sequencing and molecular diagnostics has the potential to significantly improve the ability to detect and contain pathogen transmission in advance of disease outbreaks [10]. The main goal of these technological strategies is to strategically shift the focus of surveillance from known pathogens limited to clinical to a broader exploration of potential pathogens in the environment and wildlife. It is critical to establishing a solid foundation for accurately predicting disease outbreaks, ultimately optimizing the effectiveness of preparedness and emergency response measures.

#### 2.1.1 Remote sensing

Remote sensing captures target information using various sensors without direct contact with the target. It primarily detects electromagnetic radiation in various ranges of the electromagnetic spectrum using sensors mounted on aircraft and satellite platforms to obtain information about the object being measured [11]. With the development of various remote sensing technologies such as optical, infrared and microwave technologies, the spatial, spectral, temporal, and radiometric resolution of remote sensing has been continuously improved. Remote sensing is widely used in areas such as resource investigation, environmental monitoring, weather forecasting, marine monitoring, and land management. It is also being developed as a continuous Earth observation system that supports geoscientific research and global change monitoring. Remote sensing and satellite imagery are important tools for zoonotic disease monitoring and control. These technologies provide a comprehensive overview of the environment in real time and allow researchers and health authorities to track the movement and distribution of disease vectors. In addition, satellite imagery provides a bird's eye view of the Earth's surface, allowing for mapping disease spread and identifying hotspots for potential disease outbreaks [12].

Remote sensing, as a means of obtaining biodiversity information, has developed rapidly in the field of biodiversity in recent years. Its characteristics such as wide coverage, sequence, and repeatability make it a great advantage in large-scale biodiversity monitoring, mapping, and assessment [13]. The main advantages of remote sensing as a biodiversity information source are its relatively low cost, good data consistency, and capacity for timely and regular updates [14]. The rapid development of satellite and sensor technology and the emergence of high-resolution images have significantly improved the practical application of remote sensing [15]. In recent years, the use of remote sensing technology to study biodiversity has gradually increased, and remote sensing has been applied to study the diversity of various ecosystems, from plants [16] to birds [17], from mammals [18] to butterflies [19]. By integrating remote sensing technologies with geographic information systems (GIS), China is now creating detailed habitat maps that delineate the boundaries of individual ecosystems and landscape features.

#### 2.1.2 Metagenome analysis

**Fig. 1:**
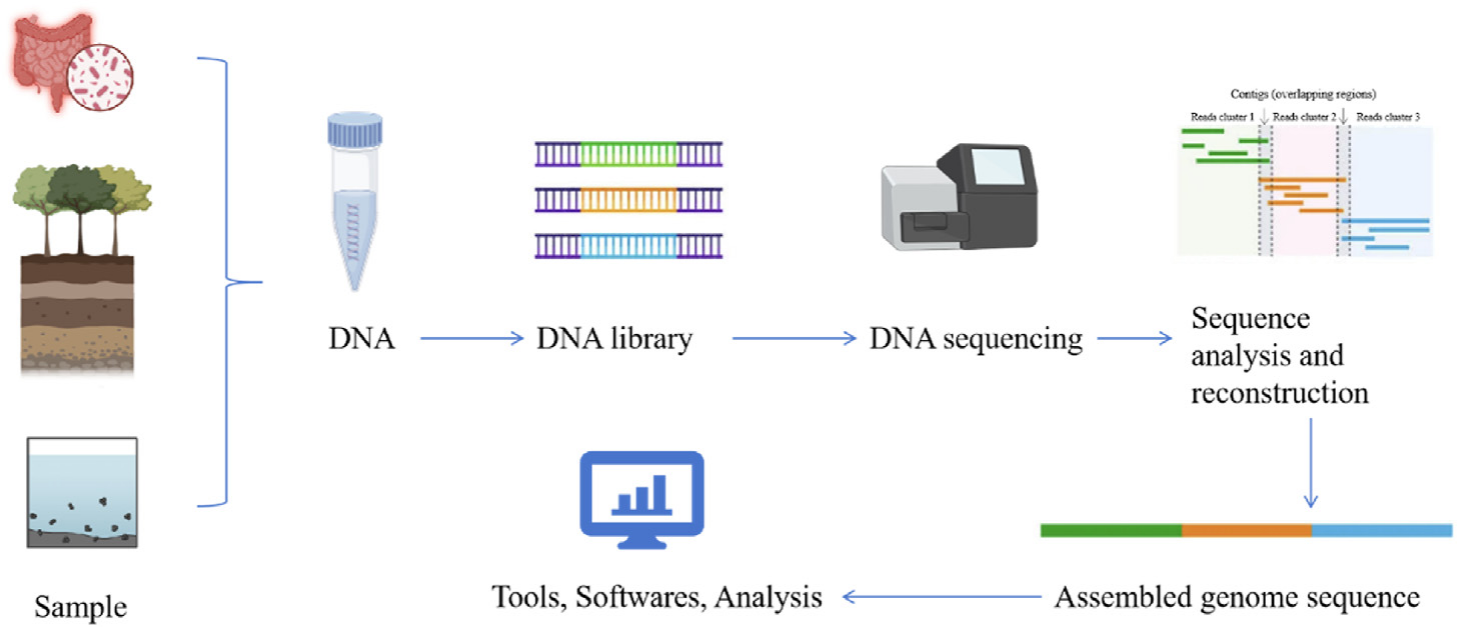
The flowchart of metagenome analysis.

Metagenomics, originally proposed by Handelsman, refers to a new microbial research method for sequencing and functional gene screening of the entire genome of all microorganisms within a specific environment [20]. Unlike traditional method, it does not need to isolate and culture microorganisms; instead, it enables direct analysis of the entire DNA of microorganisms, providing insights into the genetic, functional, ecological characteristics, and other relevant information of communities [21]. The basic process of the metagenomic genome is illustrated in Fig. 1, which consists of five steps: genome enrichment, genomic DNA extraction, metagenomic DNA library construction, target gene screening, and product activity expression [22]. By taking samples from various sources such as the human gut, the environment, soil and water, and then extracting the DNA, this technology provides a wealth of information for the control of infectious diseases. For example, gut microbiota provides clues to human health, soil is a microbial hotspot relevant to ecological and biogeochemical aspects, and DNA from water sources provides information on microbial composition and contaminants. With the maturity of sequencing technology, the reduction of sequencing cost, and the vigorous development of bioinformatics, metagenomic sequencing technology has been successfully applied to infectious diseases. The specific methodology employed in the field of infectious diseases involves the construction and screening of metagenomic libraries following sample collection. Subsequently, data statistical analysis and interpretation can be conducted after DNA sequencing and recombination. Metagenomic sequencing is particularly valuable in clinical diagnosis and differential diagnosis of diseases. Unlike traditional diagnostic methods, metagenomic sequencing technology can directly sequence cultures or samples, which greatly reduces the time of diagnosis. Metagenomic sequencing has certain advantages in the sensitivity and specificity of diagnosing EIDs.

Compared with the conventional approaches, the utilization of next-generation sequencing (NGS) technology, also known as metagenomics sequencing (mNGS), in combination with bioinformatics, has enabled researchers to identify pathogenic microorganisms more rapidly and accurately. Clinical mNGS has proven to be an effective diagnostic tool for infectious diseases due to its ability to detect a large range of pathogens. Recent advances in technology have made it an invaluable diagnostic tool for clinicians. mNGS is particularly useful for detecting infectious agents in cases of encephalitis and meningitis. For example, by utilizing mNGS, infectious diseases caused by pathogens that are less frequently encountered, such as *Angiostrongylus cantonensis*, can be accurately diagnosed, which in turn results in improved patient outcomes and care. A single mNGS assay can also identify the comprehensive spectrum of potential causes, including viral, bacterial, fungal, and parasitic pathogens, showcasing its utility in diagnosing complex central nervous system infections. Clinical mNGS has also shown promise in diagnosing various infectious diseases, including tuberculosis, HIV infection, and co-infections [23].

#### 2.1.3 Molecular diagnosis

**Fig. 2:**
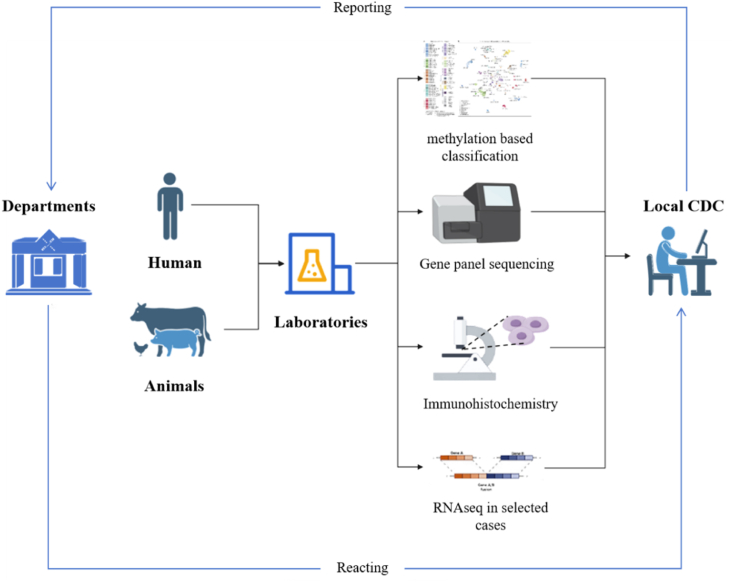
The flowchart of molecular diagnosis strategies in China. Abbreviation: CDC, center for disease control and prevention.

Molecular diagnostics is an important diagnostic method in clinical studies. Immediate testing is portable, rapid, accurate and low-cost, which helps to find the source of infection in time and control the spread of emerging infectious diseases [24]. In recent years, the development and application of molecular diagnostic techniques has triggered a revolution in the diagnosis and monitoring of infectious diseases [25]. In most conventional laboratories, microbial phenotypic characteristics, such as chromatographic, bacteriophage and protein profiles, as well as bio-typing and susceptibility tests are used for identification and differentiation. Clinical laboratories are increasingly using nucleic acid techniques, including PCR, plasmid profiling and various restriction fragment length polymorphism approaches. Rapid identification of fastidious or non-culturable bacteria is made possible by PCR-based methods, which can identify disease pathogens directly from clinical samples without the need for culture. In addition, the pathogen can be identified and better characterized by sequence analysis of the amplified microbial DNA. It has been shown that subspecies variation determined by different methods plays an important role in the prognosis of some diseases. The determination of viral load and the direct identification of genes or gene mutations that cause drug resistance are two other important developments. These technologies will become more accessible as automation and user-friendly software become increasingly accessible [26]. Molecular diagnostics has found wide application in life sciences, food safety, environmental monitoring and clinical diagnosis. Especially, in the context of the global spread of the novel coronavirus, the new technologies of the clustered regularly interspaced short palindromic repeats/CRISPR associate (CRISPR/Cas) system have initiated a rapid development in the field of molecular diagnostics, highlighting its unique advantages. Diagnostic laboratories across China are conducting rapid testing and confirmatory diagnostics to identify the pathogens responsible for livestock diseases. These include molecular techniques such as PCR, serology tests and histopathology examinations. These molecular diagnostic techniques enable the authorities to detect outbreaks of infectious diseases more quickly and take appropriate measures. With the development and effective use of these molecular technologies by local laboratories, a detection-reaction system has emerged in China, as shown in Fig. 2.

### 2.2 Surveillance and early warning system

In communal areas of China, the systematic observation, identification, and reporting of infectious diseases are collectively known as infectious disease surveillance. The current surveillance system is predominantly founded on case diagnosis, with syndromic surveillance also being incorporated. To improve the system, the Chinese government has allocated significant resources to implement new strategies, including the development of a real-time surveillance system as part of infectious disease surveillance. This strategy can serve as a model for global surveillance and response to infectious disease threats. This process is essential for the early detection of disease outbreaks, monitoring of disease trends, and implementing timely public health measures [27]. Such surveillance plays a critical role in protecting public health and improving the overall response to infectious diseases in nearby cities and neighborhoods [28]. Infectious disease surveillance involves local health departments, community health centers, and sometimes volunteers or community health workers who report suspected cases to higher health authorities [27]. The primary objective is to quickly identify and control infectious disease threats to protect public health and prevent further transmission. Given the significant impact of zoonotic diseases on public health, China is also conducting surveillance of animal populations. This includes monitoring livestock, poultry, wildlife and domestic animals for signs of infectious diseases. Surveillance measures may include regular testing, epidemiological investigations and tracking the trade and movement of animals. In refining early warning for livestock and wildlife, although Chinese standards are similar to international standards, some have been adapted to domestic conditions. In particular, a surveillance and early warning system with Chinese characteristics has been established, exemplified by the efficient networking of government, society and the public. In addition, environmental factors can influence the transmission of infectious diseases. To address this, China conducts environmental surveillance to monitor indicators such as water quality, air pollution and vector populations (e.g. mosquitoes and ticks). The integrated surveillance system for EIDs in China includes several components, including climate and ecosystem surveillance, wildlife surveillance, environmental assessment and human health surveillance, which are explained in more details below.

#### 2.2.1 Environmental monitoring and ecological surveys

Environmental monitoring and ecological surveys provide a decision-making basis for the rational use of resources and the prevention and control of diseases [29]. Environmental monitoring is widely used in disease outbreak detection and containment, and serves as a critical indicator in early warning systems and source attribution. Close monitoring of climate parameters (e.g. sea surface temperature rise and prolonged rainfall) as well as vegetation and soil indices is crucial to assess changes that could contribute to infectious disease outbreaks. The use of such data in predictive mathematical modeling could lead to early warnings to inform public health measures to contain epidemics. China conducts environmental monitoring to assess the quality of air, water, soil, and biodiversity. Environmental monitoring stations collect samples and data on pollutants, contaminants, habitat degradation, and overall ecosystem health. These efforts are crucial for identifying environmental risk factors associated with disease emergence and guiding interventions to mitigate environmental threats.

Monitoring ecosystems and biodiversity is essential for understanding ecological dynamics. This involves various tasks, including ecological assessments, species monitoring, habitat mapping and biodiversity surveys. China aims to improve its understanding of eco-health by systematically collecting and analyzing data on the composition, structure and functioning of ecosystems. This approach facilitates the development of new collaborations between human, animal and environmental health professionals and promotes global health initiatives. In addition, the project aims to deepen our understanding of the transmission of viral diseases by wildlife and develop effective strategies to prevent and manage emerging disease threats. Through the application of various biodiversity monitoring methods and the establishment of robust systems, China has conducted comprehensive biodiversity surveys to document the diversity and abundance of plant and animal species in different environments. These surveys include field trips, sampling and biodiversity inventories to record the diversity of species found in different locations.

To understand the diversity of ecosystems and assess species conservation, it is essential to map the distribution and characteristics of different habitat types. This approach provides valuable insights into population trends, distribution patterns and ecological interactions, which are crucial for the ongoing monitoring of key species. A variety of monitoring methods are used in China, such as camera traps, acoustic monitoring and citizen science, to track the abundance and behavior of target species. Ecological assessments also consider habitat quality, ecosystem services and resilience to environmental stressors when assessing ecosystem health and functioning. These assessments help prioritize conservation efforts, identify ecological hotspots, and evaluate the impact of human activities [30].

Wildlife surveys are systematic studies of animal species in their natural habitats. Conducted by field researchers and experienced biologists, these surveys use special methods to collect data on habitat use, behavior, abundance and distribution. To gain a deeper understanding of the trends and patterns of biodiversity in different ecosystems, surveys may focus on specific taxa or cover a broad range of species.

In China, the camera trap is a widely used monitoring tool for various species in different habitat types. This method offers significant advantages over traditional trapping techniques, such as pitfall traps, as cameras can remain in the field for longer periods of time [31]. Camera trapping enables the surveillance of diverse wildlife species, populations, and tracks, serving as a means to monitor their status and providing a foundation for infectious disease prevention [32]. Acoustic monitoring complements camera traps by recording and analyzing the sounds of wildlife. Acoustic data can help understand the variables that influence transmission risk, including spatial and temporal occupancy by humans and wildlife and changes in habitat quality. Using the acoustic data, researchers can identify species, monitor reproductive activity and detect changes in species composition and diversity. Genetic analysis methods such as DNA barcoding and population genetics are also used to assess genetic diversity, population structure and gene flow between animal groups. By analyzing genetic markers and extracting DNA from tissue samples such as hair, feathers or excrement, researchers can uncover links between individuals and communities [33]. Biodiversity monitoring programs in China are essential for assessing the status and trends of wildlife populations, species distribution and ecological diversity. By combining surveillance and data analysis techniques, these programs provide valuable insights for conservation planning, habitat management and species protection, contributing to the sustainable management of biodiversity resources and the conservation of natural heritage.

#### 2.2.2 Surveillance network of wildlife, livestock, and vectors

China operates a comprehensive wildlife surveillance system to monitor the health status and population dynamics of various wildlife species at the community level. This system is essential for biodiversity conservation, disease control and ecological management. The national prevention and control strategy includes the surveillance of 16 priority domestic animal diseases and 13 foreign animal diseases that require special precautions. Wildlife disease surveillance in China is an essential part of monitoring public health risks, protecting wildlife populations and preventing the spread of infectious diseases between animals and humans. The country actively monitors zoonotic diseases, i.e. those that can be transmitted from animals to humans, as more than 75 % of emerging and re-emerging infectious diseases are zoonoses, which have a significant impact on global health and the economy [34]. Surveillance efforts focus on identifying potential reservoirs of zoonotic pathogens in wildlife and assessing the risk of transmission to humans. The Chinese wildlife health authorities collect samples from a variety of animal species, including mammals, birds and reptiles, for laboratory testing. These samples, such as blood, tissue, feces, saliva, and nasal swabs, are analyzed using molecular, serological and microbiological methods to detect bacteria, antibodies, or genetic material associated with infectious diseases. To increase the effectiveness of wildlife disease surveillance and response, China has established partnerships and surveillance networks involving government agencies, universities, research institutions, and conservation organizations. These networks facilitate data sharing, collaboration and capacity building [35]. Epidemiological studies are conducted to identify the pathogen, elucidate transmission mechanisms, and assess the impact on ecosystem dynamics and wildlife health during outbreaks or unusual disease episodes. Epidemiologists and veterinarians conduct field surveys, necropsies, and molecular studies to investigate disease outbreaks and implement control measures to reduce transmission. Research in disease ecology, conservation biology and animal ecology in China is critical to improving our understanding of wildlife health and management. Through extensive research projects, China aims to clarify the complex relationships between wildlife, diseases and ecosystems, which is critical for developing targeted surveillance and intervention strategies that mitigate risks to wildlife and human health. In addition, the data collected through wildlife monitoring projects is a valuable resource for research and publication. Researchers can analyze this surveillance data to examine various indicators, including disease dynamics, population trends and overall ecosystem health. In addition, these findings support evidence-based decision-making in government agencies.

Zoonotic diseases in livestock across China pose a significant threat to human health, as these infections can easily be transmitted from animals to humans. Approximately 60 % of pathogens responsible for human diseases originate from animals [36]. Effective management of these disease requires coordinated efforts in surveillance, prevention, and control. The classification for the prevention and control of zoonotic diseases in livestock is summarized in Table 1. It includes three categories: “Key Prevention” with eight diseases, “Routine Prevention” having 14 diseases, and “Foreign Prevention” consisting of two diseases. This classification offers a structured way to prioritize and allocate resources for the prevention and control of livestock EIDs. Different categories have different objectives, indicating different strategies. “Key Prevention” diseases require a long-term and comprehensive strategy, while “Routine Prevention” diseases suggest a more maintenance-oriented approach. The selection of diseases within each category reflects the current epidemiological situation and concerns. Currently, at the community level, relevant departments strictly follow provincial and municipal guidelines, and the local animal disease prevention and control centers regularly conduct animal disease surveillance. The priority diseases to be monitored include foot-and-mouth disease, swine fever, avian influenza, and brucellosis.

**Table 1:** Classification for the prevention and control of EIDs in livestock.

Disease category	Diseases	Prevention and control objectives
Key prevention (8)	Highly pathogenic avian influenza, brucellosis, bovine tuberculosis, rabies, anthrax, echinococcosis, schistosomiasis japonica, glanders	Effectively reduce the incidence rates of diseases such echinococcosis, bovine tuberculosis, and brucellosis while controlling their prevalence; continue to eradicate glanders, control highly pathogenic avian influenza, prevent anthrax outbreaks, eradicate schistosomiasis japonica, and eventually eradicate canine-to-human rabies
Routine Prevention (14)	Toxoplasmosis, leptospirosis, salmonellosis, Japanese encephalitis (epidemic encephalitis B), *streptococcus suis* type Ⅱ infection, trichinellosis, cysticercosis, listeriosis, nocardiosis, fasciolopsiasis, psittacosis, Q fever, leishmaniasis, clonorchiasis	Keep the prevalence rates stable at a low level
Foreign Prevention (2)	Bovine spongiform encephalopathy, Nipah virus encephalitis	Effectively reduce the risk of introduction and spread of foreign diseases

Note: The numbers in parentheses represent the number of diseases under the corresponding category. Abbreviation: EIDs, emerging infectious diseases.

In China, various vector-borne diseases have spread in different regions and pose a major public health challenge, which is closely related to meteorological factors and weather events [37]. The country is now monitoring vectors such as mosquitoes, ticks and rodents to assess their distribution, abundance and vector competence. By using climate and environmental data, predictive models can be used to forecast changes in vector distribution and disease transmission under different climate change scenarios. Policy makers and health authorities can use these models to predict future disease risks and develop adaptation strategies to mitigate the impact of climate change on vector-borne diseases. Surveillance data is used to track vector-borne diseases such as Lyme disease, dengue fever and malaria and to assess the impact of environmental changes on vector populations. Vector surveillance is closely linked to disease surveillance, prompting authorities to monitor both human and animal cases of vector-borne diseases. This tracking enables the identification of areas with active vector transmission and the evaluation of the effectiveness of control measures. In addition, integrated surveillance systems facilitate early detection of disease outbreaks and enable rapid intervention.

Since 2012, China's vector surveillance system has made significant progress in combating the threats posed by vectors and related infectious diseases. Major improvements include continuous improvement of vector surveillance programs, expansion of surveillance sites, direct online reporting of surveillance data, increased financial support, and significant progress in the use of surveillance information. The system focuses on vectors and related infectious diseases and prioritizes surveillance, risk assessment and early warning.

#### 2.2.3 Human EIDs surveillance

To track, identify and respond to EIDs outbreaks in humans, China has established a comprehensive surveillance system at the community level with a list of contagious diseases that must be reported to the health authorities as soon as they are diagnosed. The legal reporting framework for infectious diseases is divided into three categories: A, B and C, comprising a total of 41 different diseases (Table 2). Category A infectious diseases include plague and cholera; category B includes 28 diseases such as tuberculosis, Mpox, rabies, COVID-19, and dengue fever; category C consists 11 diseases, including leprosy, filariasis, epidemic mumps and hand-foot-and-mouth disease. In addition, the framework also includes other infectious diseases designated by the National Health and Family Planning Commission for the management of category B and C, as well as those requiring emergency surveillance and notification as part of category A management. Healthcare facilities and service providers must use electronic reporting systems to report suspected and confirmed cases of notifiable diseases to health authorities. These reports facilitate the early detection of disease outbreaks, enable rapid responses and the implementation of effective control measures.

**Table 2:** Classification and reporting mechanism of EIDs.

Disease type	Diseases	Reporting
Category A (2)	Plague, cholera	In urban areas, the reporting of EIDs must occur within 6 h of identification, whereas in rural areas, the report should be completed within 12 h
Category B (28)	SARS, viral hepatitis, avian influenza A (H5N1), epidemic hemorrhagic fever, Japanese encephalitis, anthrax, tuberculosis, meningococcal meningitis, diphtheria, scarlet fever, gonorrhea, leptospirosis, malaria, COVID-19, AIDS, polio, measles, rabies, dengue fever, bacterial and amebic dysentery, typhoid and paratyphoid fever, pertussis, tetanus in newborns, brucellosis, syphilis, schistosomiasis, avian influenza A (H7N9), Mpox	Regardless of whether in urban or rural settings, reporting through the web-based infectious disease surveillance information system is mandated within 24 h following diagnosis; for diseases classified as category A, such as pulmonary anthrax and SARS, reporting must occur within a 2-h timeframe
Category C (11)	Influenza, rubella, leprosy, Kala-azar, filariasis, hand, foot, and mouth disease, epidemic parotitis, acute hemorrhagic conjunctivitis, epidemic and endemic typhus, echinococcosis, other EIDs	It is required to report via the system within 24 h after discovery

Note: The numbers in parentheses represent the number of diseases under the corresponding category. Abbreviations: EIDs, emerging infectious diseases; SARS, severe acute respiratory syndrome; AIDS, acquired immunodeficiency syndrome.

China has established a laboratory network to monitor new EID cases. These laboratories utilize various techniques, including diagnostic tests, pathogen identification, and gene sequencing, to characterize pathogens. Laboratory surveillance provides essential data for disease monitoring and identification. In addition, syndromic surveillance systems are used to track population trends in disease and healthcare seeking behavior. This type of surveillance collects information from various sources, such as pharmacy sales, emergency room visits and outpatient consultations. Through early detection of unusual disease patterns or clusters, syndromic surveillance plays a critical role in signaling potential infectious disease outbreaks.

**Fig. 3:**
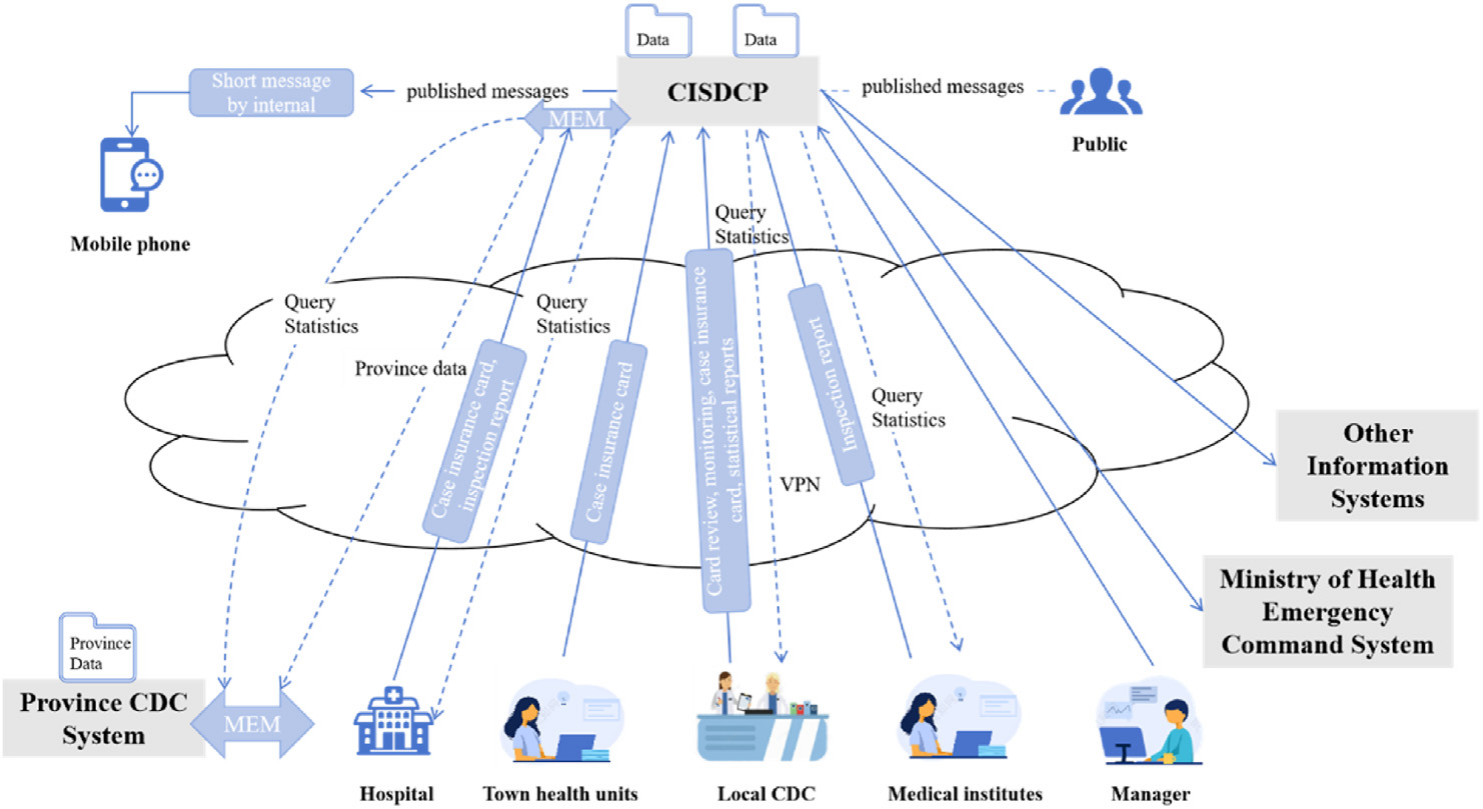
Web-based reporting system for EIDs in China. Abbreviations: EIDs, emerging infectious diseases; CISDCP, Chinese Information System for Disease Control and Prevention; VPN, virtual private network; CDC, center for disease control and prevention.

With the rapid development of information technology, the surveillance and reporting system for EIDs in China at community level has evolved significantly [38]. Since 2004, China has developed a web-based reporting system (Fig. 3), which is fast, accurate and has a wide coverage area. This system enables real-time data updates and facilitates information sharing between central and local authorities, which has a significant impact on the early detection, rapid response and effective control of EIDs. In the outbreaks, the relevant authorities can quickly access accurate information and take timely prevention and control strategies to mitigate the social and economic impact. The web-based reporting system represents a significant advance in EIDs surveillance. It has improved both the accuracy and timeliness of surveillance and provides a solid data basis for decision-making, contributing to the development of a more resilient surveillance and early warning system.

## 3 Gaps, challenges, and opportunities

### 3.1 Integration and coordination of surveillance system

China needs to improve the integration and coordination of its human health, animal health and environmental health sectors. Fragmented systems and isolated approaches can hinder timely detection and response to EIDs that emerge at the human–animal–environment interface.

The lack of communication and collaboration between the different surveillance systems can lead to various problems. For example, inadequate data sharing can create information silos that prevent competent authorities from promptly accessing important information collected by other agencies [39]. This limitation can affect their ability to fully understand and effectively respond to potential health threats. When systems operate independently, it can also lead to unnecessary duplication of effort, waste of time, resources and manpower, especially when multiple departments are collecting similar or redundant data.

Effective monitoring depends on the timely sharing of data and information across different sectors and geographical areas [40]. However, there are still problems with data exchange protocols, interoperability of information systems and communication channels between relevant authorities. Improving data sharing mechanisms and enhancing communication between stakeholders is crucial for early detection and response to EIDs.

### 3.2 Systematic surveillance building

Current surveillance systems often fail to cover all geographical areas or relevant species, leading to detection gaps. Many local health authorities operate independently and may not have consistent surveillance capacity, leading to under-detection, particularly in rural or remote areas [41]. Traditional surveillance systems typically evaluate the status of EIDs by considering individual factors, such as internet usage, telephone triage hotlines, drug sales and absenteeism [42]. However, this approach lacks a comprehensive, integrated perspective. While China is developing a new surveillance and early warning system for EIDs under the One Health concept, several challenges and gaps need to be further addressed.

The lack of systematic surveillance leads to significant problems, including the absence of a comprehensive perspective on health issues that often include human, animal and environmental factors. An integrated approach is essential for an effective solution. In addition, authorities may be slow to respond to potential health threats and have difficulty accessing, sharing and analyzing data in emergencies. This inefficiency can lead to a misallocation of resources. Therefore, a sound approach at the highest level and an effective institutional framework is crucial.

### 3.3 Capacity building

Limited capacity-building efforts exacerbate the challenges of anticipating and responding to emerging threats, as relevant professionals may lack the necessary skills to effectively collect and analyze data [43]. Opportunities for cross-sectoral training on the One Health approach are currently limited and inconsistent across provinces, with gaps for certain priority infectious diseases. The lack of a robust training program can affect the performance and overall effectiveness of surveillance efforts. Therefore, a comprehensive approach that includes thorough planning and sustained capacity-building initiatives is critical to establishing an effective and proactive surveillance system. In addition, investment in laboratory infrastructure and diagnostic capabilities, particularly in rural and remote areas, is crucial.

### 3.4 Research and innovation limitation

Despite significant technological advances in China today, the availability and accessibility of infrastructure in certain rural and remote areas remain limited. Challenges such as unreliable internet connectivity, inconsistent power supply and inadequate laboratory facilities can hinder the implementation of complex monitoring techniques and limit the effective use of diagnostic tools. In addition, fostering innovation in surveillance techniques, diagnostic tools and predictive modeling methods is critical to improving early detection, risk assessment and prediction of EIDs outbreaks [44]. Addressing these limitations is critical to improving the overall efficiency of the surveillance and response system.

## 4 Perspectives

### 4.1 Establish a multi-trigger integrated surveillance system

**Fig. 4:**
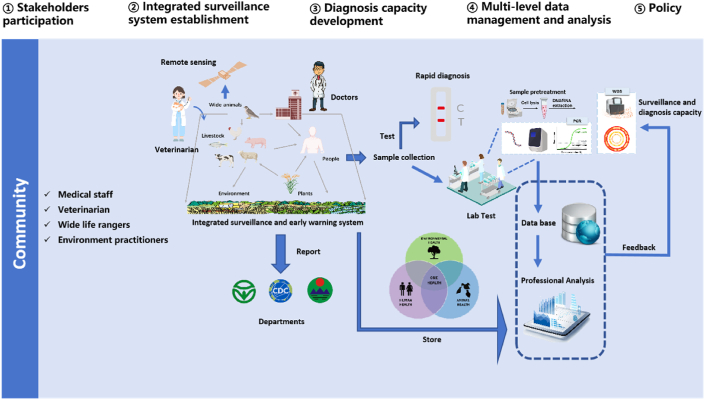
Integrated surveillance system structure in China.

The integrated surveillance and early warning system (Fig. 4) highlights the importance of multiple factors from multi-levels, which is crucial for effectively monitoring, detecting, and responding to EIDs. This strategy shifts the attention from the disease surveillance in individuals and groups to a comprehensive assessment of the various determinants, particularly at the human–animal–environment interface under the One Health concept. The comprehensive surveillance and early warning system will facilitate the realization of effective prevention and response to EIDs by expanding both the scope and depth of traditional disease early warning systems.

To further improve the integrated multi-trigger surveillance system of EIDs in China at community level, it is essential to strengthen infectious disease surveillance and expand surveillance channels. This includes optimizing the existing epidemic surveillance and reporting system to improve the surveillance of clinical manifestations, pathogenic microorganisms, vectors, and environmental factors related to EIDs. In addition, refining the processes for risk assessment and dissemination of early warning information, standardizing the assessment and warning procedures, and advancing laboratory techniques are all critical steps.

The pathogen escape routes within the “environment-wildlife/habitat-human” chain should be the focus of integrated multi-trigger surveillance systems. Efforts will be made to integrate the existing human disease surveillance system, livestock disease surveillance system, wildlife epidemic surveillance system, and ecological surveillance system into a unified One Health-based surveillance system.

### 4.2 Strengthen multi-sectoral and interdisciplinary cooperation

To enhance multi-sectoral and interdisciplinary collaboration at community level under the One Health concept, a comprehensive and inclusive framework is imperative, bridging human, animal, and ecological health domains. Operationalizing this collaboration involves establishing robust cross-sectoral communication channels to facilitate seamless information dissemination and integrated decision-making among health departments, agricultural sectors, environmental agencies, research institutions, and policymakers. Fostering cultural competence and respect among stakeholders is crucial for establishing trust and facilitating efficient collaboration. In addition, cohesive monitoring and surveillance mechanisms are essential for tracking health trends across species and environments.

### 4.3 Efficient data sharing and analysis system

The integrated EIDs surveillance system connects disparate facilities such as medical facilities, disease control centers and community health networks, ensuring data interoperability and promoting seamless coordination of operational workflows. This implementation leverages the wide reach of the healthcare network and accesses surveillance data stored in the hospital information systems (HIS), laboratory information systems (LIS) and picture archiving and communication systems (PACS) of the medical facilities.

This integration not only diversifies data sources, but also emphasizes the importance of data gateways and improves responsiveness through multi-level triggering mechanisms. Designed to extend its monitoring reach to different levels and types of medical and healthcare facilities, including pharmacies, private clinics and workplaces, the system is based on the principles of multi-point triggering and multi-channel monitoring. By incorporating cutting-edge technologies and relentless innovation, the system will make an important contribution to infectious disease surveillance and response capabilities, increasing efficiency and practicality. For example, surveillance systems can be strengthened by big-data streams, including electronic health (e-health) patient records, and non-traditional digital data sources, such as social media, Internet, mobile phones, and remote sensing [45]. E-health patient records contain a large amount of detailed clinical information about patients’ medical history, symptoms, diagnoses and treatment methods. These records, when integrated into the surveillance framework, can provide valuable insights into disease prevalence and patterns. In addition, non-traditional digital data sources play an equally important role. Social media platforms, with their billions of active users worldwide, can provide real-time information on public health issues. In the context of the increasing number of global infectious disease outbreaks, which have led to several public health emergencies of international concern and imposed an enormous impact on population health, the economy, and social development, the importance of enhancing early surveillance capabilities has been emphasized. This is essential for constructing a common biosecurity shield for the global community in pursuit of health for all [39].

## 5 Conclusions

This review systematically summarizes the integrated surveillance and early warning system for EIDs at the community level in China. It covers the current status of EIDs surveillance and early warning systems, including the technique employed for human, environmental and animal EIDs surveillance systems at the community level in China. Our analysis reveals the shortcomings in EIDs monitoring and early warning systems. To address these gaps, we advocate for establishing an integrated monitoring system with multiple triggers through multi-sectoral and interdisciplinary collaboration. This approach aims to create an efficient framework for sharing, managing, and analyzing data. By providing guidance for formulating more effective disease prevention and control strategies, our findings and guidelines will strengthen the resilience of communities in the face of future EIDs challenges.

## CRediT authorship contribution statement

**Chenjia Zhou:** Writing – review & editing, Writing – original draft, Methodology, Investigation, Conceptualization. **Suping Wang:** Writing – review & editing, Writing – original draft, Investigation. **Chenxi Wang:** Writing – review & editing, Writing – original draft, Methodology, Conceptualization. **Ne Qiang:** Writing – review & editing, Writing – original draft, Methodology, Investigation, Conceptualization. **Leshan Xiu:** Writing – review & editing, Writing – original draft, Investigation. **Qinqin Hu:** Writing – review & editing, Writing – original draft, Investigation. **Wenyu Wu:** Writing – review & editing, Writing – original draft, Investigation. **Xiaoxi Zhang:** Writing – review & editing, Investigation. **Lefei Han:** Writing – review & editing, Investigation. **Xinyu Feng:** Writing – review & editing, Investigation. **Zelin Zhu:** Writing – review & editing, Investigation. **Leilei Shi:** Writing – review & editing, Investigation. **Peng Zhang:** Writing – review & editing, Investigation. **Kun Yin:** Writing – review & editing, Writing – original draft, Investigation, Conceptualization.

## Funding

This work was supported by the Hainan Province Science and Technology Special Fund (grant number ZDYF2022SHFZ321); National Natural Science Foundation of China (grant numbers 22104090, 82102184) and the Natural Science Foundation of Shanghai (grant numbers 22ZR1436200, 23ZR1436800), Shanghai Municipal Health Commission of China (grant number 2024QN083) and Shanghai Jiao Tong University School of Medicine (grant number WK2406).

## Declaration of competing interest

The authors declare that they have no known competing financial interests or personal relationships that could have appeared to influence the work reported in this paper.
